# Recent trends in cervical cancer mortality in Britain and Ireland: the case for population-based cervical cancer screening

**DOI:** 10.1038/sj.bjc.6602236

**Published:** 2004-11-16

**Authors:** H Comber, A Gavin

**Affiliations:** 1 National Cancer Registry, Elm Court, Boreenmanna Road, Cork, Ireland; 2 Northern Ireland Cancer Registry, Department of Epidemiology and Public Health, Queens University of Belfast, Mulhouse Building, Grosvenor Road, Belfast BT12 6BJ, UK

**Keywords:** cervix, mortality, trends, screening

## Abstract

This study used published mortality data and regression techniques to look at time trends in cervical cancer mortality between 1970 and 2000 in the UK and the Republic of Ireland. Mortality from cancer of the cervix has been declining in the UK for at least the past 30 years. The rate of decrease has been greatest in England, Wales and Scotland and has accelerated in these countries since the reorganisation of screening services in the late 1980s. Mortality in Northern Ireland is also decreasing, but at a lesser rate and without significant change over the same period. In contrast, cervical cancer mortality in the Irish Republic, which, unlike the UK, does not have comprehensive population-based screening, has been increasing by an average of 1.5% per year since 1978. The mortality rate, which was half of that in the UK in the late 1970s, now exceeds that in any of the region of the UK. The absence of population-based screening for cervical cancer in the Republic of Ireland is the most plausible explanation for these differences in trend.

Population-based cervical screening programmes have been shown to be effective in reducing morbidity and mortality from cervical cancer (Laara *et al*, 1987; Anderson *et al*, 1988; Hakama and Louhivuori, 1988). Different mortality trends in Nordic countries have been ascribed to the ways in which cervical screening was delivered (Laara *et al*, 1987; Hakama and Louhivuori, 1988). While Iceland, Finland, Sweden and parts of Denmark achieved almost complete coverage of the target populations, resulting in sharp falls in incidence and mortality, Norway did not have an organised screening programme and the risk of invasive cancer continued to rise into the late 1970s.

Opportunistic screening, with recall of those who previously had a smear, began in Britain in 1964 (Cancer Research UK, 2003). However, in England prior to 1988, at least two-thirds of patients with invasive cancer had never been screened and in those aged over 40 years (70% of cases), 90% had never been screened (Cancer Research UK, 1994). In the UK, the 1987 recommendation of the Intercollegiate Working Party (Intercollegiate Working Party on Cervical Cytology Screening, 1987) recommended population screening and by 1991 all District Health Authorities operated population screening on either a 3 or 5 yearly basis. Coverage of women in the target age range had increased from 43% in 1988/9 to 83% in 1992/3 (Cervical Screening Programme England 2001–02, 2002). In contrast, in the Republic of Ireland, although there is a high level of opportunistic screening (Department of Health and Children, 2003), the only population-based screening is a small pilot project in the MidWestern Health Board, covering 9% of the national population (Irish Cervical Screening Project, 2003).

This study compares trends in cervical cancer mortality between 1970 and 2001 in Ireland, Northern Ireland, Scotland, England and Wales, and investigates differences in rates and trends, which might be attributable to population-based screening.

## MATERIALS AND METHODS

Official mortality statistics were examined for all the regions from 1971 to the most recent available date. The JoinPoint Regression Programme, developed at the National Cancer Institute (JoinPoint Regression Program, 2000), was used to fit linear regression lines to the age-standardised (World population) mortality rates and to identify points of inflection. Variances for each point were calculated from the number of observed deaths.

## RESULTS

In 1971, mortality from cancer of the cervix in Britain was much higher than in Ireland and slightly higher in Northern Ireland than in the Republic (Table 1

**Table 1:** Average annual age-standardised mortality from cancer of the cervix in Britain and Ireland (1971–2000)

	**Annual age-standardised mortality rates (number of deaths)**			
**Year of death**	**England and Wales**	**N. Ireland**	**Scotland**	**Ireland**
*All ages*				
1971–75	5.7 (10993)	4.6 (227)	5.7 (1151)	3.7 (344)
1976–80	5.4 (10659)	3.3 (172)	5.3 (1053)	2.8 (270)
1981–85	5.0 (9849)	3.6 (182)	5.2 (1056)	2.9 (300)
1986–90	4.7 (9450)	3.4 (184)	4.7 (945)	3.1 (331)
1991–95	3.5 (7509)	2.9 (161)	4.0 (829)	3.0 (288)
1996–99	2.7 (4805)	2.8 (140)	3.0 (549)	3.3 (335)
2000–01	1.6 (2145)	2.3 (54)	2.6 (230)	3.9 (322)
				
*Women aged 20–34 years*				
1971–75	1.1 (271)	0.4 (3)	0.9 (22)	0.9 (12)
1976–80	1.6 (437)	1.3 (10)	1.3 (36)	0.6 (11)
1981–85	2.0 (576)	1.5 (13)	1.9 (57)	1.1 (23)
1986–90	2.0 (582)	1.1 (10)	2.3 (69)	0.7 (15)
1991–95	1.4 (445)	0.7 (7)	1.3 (40)	0.7 (17)
1996–99	1.1 (272)	1.4 (12)	1.0 (27)	1.0 (15)
2000–01	0.9 (120)	0.4 (2)	1.1 (12)	1.0 (19)
				
*Women aged 35 years and over*				
1971–75	14.3 (10722)	11.8 (224)	14.4 (1129)	9.3 (332)
1976–80	13.4 (10222)	7.9 (162)	13.2 (1017)	7.1 (259)
1981–85	11.9 (9273)	8.7 (169)	12.5 (999)	6.9 (277)
1986–90	11.3 (8868)	8.2 (174)	11.1 (876)	7.8 (316)
1991–95	8.4 (7064)	7.2 (154)	9.7 (789)	7.7 (271)
1996–99	6.4 (4533)	6.5 (128)	7.3 (522)	8.4 (320)
2000–01	4.0 (2025)	5.7 (52)	6.1 (218)	8.4 (303)

). However, between 1971 and 2000, mortality in Britain dropped dramatically, especially after 1989, and that in Northern Ireland more slowly, while that in Ireland did not. As a result, by 2000, cervical cancer mortality in the Republic of Ireland exceeded that in all the other regions. In England, Wales and Scotland, the mortality rate for women age 20–34 years halved between 1986 and 2000–2001. No trend was apparent in Ireland, and the numbers were too small to draw any conclusion concerning Northern Ireland. However, the numbers in this age group were too small to have any effect on overall trends.

**Figure 1:**
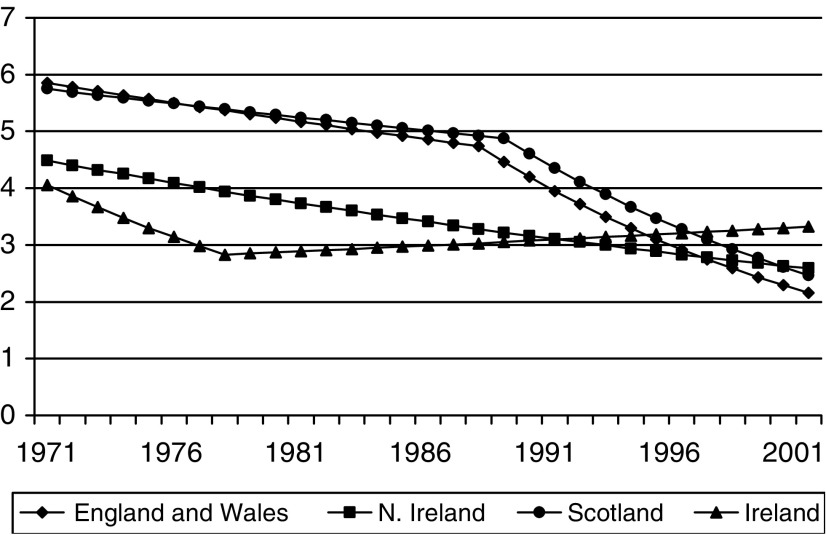
Modelled trends in cervical cancer mortality in Britain and Ireland (1971–2001).

JoinPoint modelling (Figure 1

) showed the trends for England, Wales and Scotland to be almost identical. In England and Wales, mortality rates fell slowly before 1988, with an estimated annual percentage decrease of 1.0% with 95% confidence intervals (CI) of 0.7–1.2%, but after 1988 the rate of decrease was much greater, 4.6% per annum (95% CI 4.2–5.0%). In Scotland, the rate of decrease went from 1.0% per annum (95% CI 0.4–1.6%) before 1989 to 4.2% (2.9–5.5%) after that date. In Northern Ireland, there was a small but steady decrease in mortality over the period 1971–2000, with an annual rate of decrease of 1.8% (95% CI 1.0–2.6%), but no observable point of inflection.

In the Republic of Ireland, the pattern was quite different. Prior to 1978, cervical cancer mortality fell by an estimated 3.9% per year (95% CI 1.0–8.7%), but this trend reversed to give a steady increase of 1.5% per annum (95% CI 0.6–2.3%) up to 2001. The model suggests that this trend will continue into the near future.

## DISCUSSION

In England, Scotland and Wales, mortality from cancer of the cervix has been declining since 1970, as reported previously (Quinn *et al*, 1999). These long-term trends in death rates have brought mortality in England, Scotland and Wales below that in the Republic of Ireland for the first time. If current trends continue, mortality rates in Northern Ireland also look likely to exceed those in the rest of the UK in the near future. The reduction in deaths up to 1997 in England due to screening has been estimated at 800 (Quinn *et al*, 1999), 1300 (Sasieni and Adams, 1999) or 2000 (Quinn *et al*, 1999; Peto *et al*, 2004).

Cervical cancer mortality in Northern Ireland has historically been lower than in Britain. The impact of population-based screening in the past two decades seems lower than that in Britain and, although mortality is steadily falling, the more rapid rate of decrease seen in the rest of the UK in the 1990s has not happened in Northern Ireland. However, the data for Northern Ireland are sparse and an acceleration in the decline of mortality in the late 1980s may be concealed within the random variation. On the other hand, cervical cancer mortality in the Republic of Ireland has increased steadily throughout the 1980 and 1990s and looks like continuing this trend into this decade. As the number of deaths registered as due to cancer of the uterus (part unspecified) has remained low throughout this period (Central Statistics Office, 1974, 2002), changes in death registration practice seem an unlikely explanation for the trend. Assuming that the trend is real, there are two possible, but not mutually exclusive, explanations for the increase – the incidence of cervical cancer may be increasing or survival may be worsening. Data from the National Cancer Registry (National Cancer Registry, 2003) show that the incidence of invasive cervical cancer decreased by 1.1% annually between 1994 and 1999, but no incidence information is available for the period before 1994. It is possible, although unlikely, that the trend may have been in the opposite direction in the 1980s and 1990s. Major changes in sexual behaviour seem to have occurred during that time, as evidenced by an increase in the percentage of births outside marriage from 5% (1980) to 30% (Central Statistics Office, 2002) and in sexually transmitted infection, including a ten-fold increase in the incidence of anogenital warts between 1989 and 2000 (National Disease Surveillance Centre, 2001). However, similar changes have occurred in the UK without a corresponding increase in cervical cancer mortality (Peto *et al*, 2004).

The second possibility is of a decrease in survival, although this seems unlikely in view of the general increase in cervical cancer survival throughout Europe during this period (Coleman *et al*, 2003). The most probable explanation is that in Ireland, unlike the UK, an increase in cervical cancer incidence has not been offset by the improvement in detection of precancerous lesions, and so survival, brought about by screening (Peto *et al*, 2004). Early detection through effective screening is a major factor in improving survival. Screening activity in Ireland can be measured only through the number of smears processed in public laboratories, which was over 180 000 in 2000 (Department of Health and Children, 2003). If all women in the target group were having regular screening, this volume of activity would equate, over a 3-year period, to 61% coverage of the female population aged 25–59 years. However, no information is available on the ages of women screened, nor on their screening history, so women of the wrong age, or those with recent negative tests, may make up a significant proportion of the total.

The experience of the Nordic countries and of British Columbia (Laara *et al*, 1987; Anderson *et al*, 1988; Hakama and Louhivuori, 1988) suggests that only those with an organised population-based programme show significant decreases in mortality. Although deaths from cervical cancer are relatively infrequent in Ireland (76 per year on average; 2.2% of all cancer deaths), they are largely preventable through screening. The opportunistic screening at present carried out in Ireland appears to be having little impact on the overall rate of mortality from cervical cancer. A move from the current system to a population-based programme would not greatly increase the volume of screening while probably bringing about the type of mortality reduction seen elsewhere.

## References

[bib1] Anderson GH, Boyes DA, Benedet JL, Le Riche JC, Matisic JP, Suen KC, Worth AJ, Millner A, Bennett OM (1988) Organisation and results of the cervical cytology screening programme in British Columbia, 1955–85. BMJ (Clin Res Ed) 296: 975–978 10.1136/bmj.296.6627.975 PMC2545443 3129115

[bib2] Cancer Research Campaign (1994) Cervical Cancer Screening Factsheet 13.1

[bib3] Cancer Research UK (2003) Cancer Stats – Cervical Screening UK

[bib4] Central Statistics Office (1974) Report on Vital Statistics 1971. Dublin: Government Publications Office

[bib5] Central Statistics Office (2002) Report on Vital Statistics 2000. Dublin: Government Publications Office

[bib6] Cervical Screening Programme England 2001–02 (2002). London: Department of Health

[bib7] Coleman MP, Gatta G, Verdecchia A, Estève J, Sant M, Storm H, Allemani C, Ciccolallo L, Santaquilani M, Berrino F, the EUROCARE Working Group (2003) EUROCARE-3 summary: cancer survival in Europe at the end of the 20th century. Ann Oncol 14(Suppl 5): v128–v149 14684503 10.1093/annonc/mdg756

[bib8] Department of Health and Children, Ireland (2003) Unpublished data

[bib9] Hakama M, Louhivuori K (1988) A screening programme for cervical cancer that worked. Cancer Surv 7: 403–416 3242792

[bib10] Intercollegiate Working Party on Cervical Cytology Screening (1987) Report. London: Royal College of Obstetricians and Gynaecologists

[bib11] Irish Cervical Screening Project (2003) Annual Report 2001

[bib12] Joinpoint Regression Program, Version 2.5 (2000) National Cancer Institute

[bib13] Laara E, Day NE, Hakama M (1987) Trends in mortality from cervical cancer in the Nordic countries: association with organised screening programmes. Lancet 1: 1247–1249 2884378 10.1016/s0140-6736(87)92695-x

[bib14] National Cancer Registry (2003) Cancer in Ireland 1994–2002. Cork: National Cancer Registry

[bib15] National Disease Surveillance Centre (2001) Quarterly Report on Sexually Transmitted Infections; Quarter 4 2000 (including annual summary). Dublin: National Disease Surveillance Centre

[bib16] Peto J, Gilham C, Fletcher O, Matthews FE (2004) The cervical cancer epidemic that screening has prevented in the UK. Lancet 364: 249–256 15262102 10.1016/S0140-6736(04)16674-9

[bib17] Quinn M, Babb P, Jones J, Allen E, (on behalf of the UK Association of Cancer Registries) (1999) Effect of screening on incidence of and mortality from cancer of cervix in England: evaluation based on routinely collected statistics. BMJ 318: 904–908 10102852 10.1136/bmj.318.7188.904 PMC27810

[bib18] Sasieni P, Adams J (1999) Effect of screening on cervical cancer mortality in England and Wales: analysis of trends with an age period cohort model. BMJ 318: 1244–1245 10231253 10.1136/bmj.318.7193.1244 PMC27862

